# Distribution of *ermB*, *ermF*, *tet(W)*, and *tet(M)* Resistance Genes in the Vaginal Ecosystem of Women during Pregnancy and Puerperium

**DOI:** 10.3390/pathogens10121546

**Published:** 2021-11-26

**Authors:** Marco Severgnini, Tania Camboni, Camilla Ceccarani, Sara Morselli, Alessia Cantiani, Sara Zagonari, Giulia Patuelli, Maria Federica Pedna, Vittorio Sambri, Claudio Foschi, Clarissa Consolandi, Antonella Marangoni

**Affiliations:** 1 Institute of Biomedical Technologies, National Research Council, 20054 Segrate, Italy; marco.severgnini@itb.cnr.it (M.S.); tania.camboni@itb.cnr.it (T.C.); camilla.ceccarani@itb.cnr.it (C.C.); clarissa.consolandi@itb.cnr.it (C.C.); 2 Microbiology, Department of Experimental, Diagnostic and Specialty Medicine (DIMES), University of Bologna, 40128 Bologna, Italy; sara.morselli6@unibo.it (S.M.); alessia.cantiani@studio.unibo.it (A.C.); vittorio.sambri@unibo.it (V.S.); antonella.marangoni@unibo.it (A.M.); 3 Family Advisory Health Centres, 48121 Ravenna, Italy; sara.zagonari@auslromagna.it (S.Z.); giulia.patuelli@auslromagna.it (G.P.); 4 Unit of Microbiology, Greater Romagna Hub Laboratory, 47023 Cesena, Italy; mariafederica.pedna@auslromagna.it

**Keywords:** vaginal microbiome, resistance genes, macrolide, tetracyclines, pregnancy, women’s health

## Abstract

The inhabitants of the vaginal ecosystem can harbor genetic determinants conferring antimicrobial resistance. However, detailed data about the distribution of resistance genes in the vaginal microbiome of pregnant women are still lacking. Therefore, we assessed the presence of macrolide (i.e., *erm* genes) and tetracycline (i.e., *tet* genes) resistance markers in the vaginal environment of Caucasian women at different gestational ages. Furthermore, the detection of resistance genes was related to the composition of the vaginal microbiota. A total of 228 vaginal samples, collected at different trimesters of pregnancy or during the puerperium, were tested for the presence of *ermB*, *ermF*, *tet(W)*, and *tet(M)* by in-house end-point PCR assays. The composition of the vaginal microbiota was assessed through a microscopic evaluation (i.e., Nugent score) and by means of sequencing V3–V4 hypervariable regions of the bacterial 16 rRNA gene. Overall, the most detected resistance gene was *tet(M)* (76.7%), followed by *ermB* (55.2%). In 17% of women, mainly with a ‘normal’ vaginal microbiota, no resistance genes were found. Except for *tet(W)*, a significant correlation between the positivity of resistance genes and a dysbiotic vaginal status (i.e., bacterial vaginosis (BV)) was noticed. Indeed, samples positive for at least one resistance determinant were characterized by a decrease in *Lactobacillus* spp. and an increase of BV-related genera (*Prevotella, Gardnerella, Atopobium, Sneathia*). A high predominance of vaginal *Lactobacillus* spp. (>85%) was associated with a lower risk of *tet(W)* gene detection, whereas the presence of *Megasphaera* (>1%) increased the risk of positivity for all analyzed genes. Different types of vaginal microbiota are associated with peculiar resistance profiles, being a lactobacilli-dominated ecosystem poor in or free of resistance genes. These data could open new perspectives for promoting maternal and neonatal health.

## 1. Introduction

In healthy reproductive-aged women, the vaginal microbiome is generally dominated by members of the *Lactobacillus* genus, able to promote the maintenance of the vaginal eubiosis, preventing the colonization and growth of adverse microorganisms [1,2,3,4].

The depletion of lactobacilli, combined with the increase in different species of anaerobic bacteria, can result in the switch from a normal vaginal consortium to a polymicrobial dysbiosis known as bacterial vaginosis (BV) [5,6]. This condition is characterized by higher levels of different anaerobes, including *Gardnerella vaginalis*, *Atopobium*, *Prevotella*, *Mobiluncus*, and *Veillonella* spp. [7,8].

The composition of the vaginal microbiome can vary throughout a woman’s life in response to various factors, such as diet, hormonal levels, sexual habits, pregnancy, pharmaceutical treatments, and urogenital infections [9,10]. In particular, during pregnancy, the vaginal microbiome undergoes significant changes, with a marked decrease in overall diversity and enrichment in *Lactobacillus* spp. [11,12].

It is known that the inhabitants of the vaginal microbiome can harbor several genetic determinants conferring antimicrobial resistance [13,14]. Among them, the ribosome protection type of tetracycline (i.e., *tet* genes) and the methylase type erythromycin resistance genes (i.e., *erm* genes) are the most common [15,16,17].

The *tet(M)*, *tet(W)*, *ermB*, and *ermG* genes are most often found in the Firmicutes, and the *tet(Q)* and *ermF* genes are found widely in the Bacteroidetes [13].

Both these families of resistance genes can be linked to DNA from known conjugative transposons (mobile elements), thus favoring horizontal transfer across genus and species lines [18]. In this way, commensal microorganisms can act as reservoirs of antibiotic resistance genes that can be ultimately transferred to pathogens.

The direct detection of tetracyclines and macrolide resistance genes have been performed mainly on the human gut or oral microbiota (i.e., fecal and saliva samples) or on food-borne specimens, whereas the distribution of *tet* and *erm* genes in the vaginal ecosystem is largely unexplored [19,20,21,22,23].

A decade ago, Jeters and colleagues assessed the presence of antibiotic resistance determinants in the vaginal microbiota of two populations of primates never exposed to antibiotics, demonstrating a high prevalence of *tet(M)* and *tet(W)* genes [13].

It is well known that the vaginal bacterial composition plays a crucial role in maternal-fetal health: indeed, a condition of vaginal dysbiosis and its related bacterial taxa are associated with several pregnancy complications and preterm birth [12,24,25]. 

Moreover, during delivery, the composition of the vaginal ecosystem can affect the newborn’s health, shaping the development of the infant’s bacterial communities and modulating the risk of disease susceptibility later in life [26].

Thus, studying the distribution of macrolide and tetracycline resistance genes and their association with vaginal bacterial communities in pregnant women could open new perspectives for the management of pregnancy and the care of newborn health.

Therefore, in this study, we assessed the presence of two markers of macrolide resistance, *ermB* and *ermF*, as well as two determinants of tetracycline resistance, *tet(W)* and *tet(M)*, in the vaginal microbiota of Caucasian women at different gestational ages and during the puerperium. The detection of resistance genes was subsequently related to the characteristics of the vaginal microbiome in terms of bacterial composition obtained by sequencing of the bacterial 16S rRNA gene.

## 2. Results

### 2.1. Study Population and Samples

During the study period, a total of 228 vaginal samples were collected from 72 Caucasian women. In particular, 72 samples were collected during the first trimester of pregnancy, 63 during both the second and third trimester, and the remaining 30 during the puerperium. Over time, the drop-out of women from the study was mainly due to antibiotic treatments or Cesarean section.

At the beginning of pregnancy, the mean age of women was 31.2 ± 5.3 (range: 21–44 years), whereas the mean BMI was 23.6 ± 3.6 (range: 16.3–32.5 kg/m^2^).

Overall, based on Nugent score [27], 142 vaginal samples (62.3%) were characterized by a lactobacilli-dominated bacterial composition (Nugent score 0–3), 51 (22.4%) by an intermediate microbiota (Nugent score: 4–6), and the remaining 35 (15.3%) harbored a BV-associated microbial composition (Nugent score: 7–10). Cases of BV were mainly found during the first trimester of pregnancy (13/35; 37.1%) or during the puerperium (12/35; 34.3%) (Table S1).

### 2.2. Detection of Resistance Genes

The most detected resistance gene was *tet(M)*, with 175 cases (76.7%), followed by *ermB* (126 cases, 55.2%), *ermF* (58 cases, 25.4%), and *tet(W)* (33 cases, 14.4%). It is worth noting that in 39 women (17.1%), the majority of which belonged to the “H” group (34/39; 87.2%), no resistance genes were found.

For all the four genes analyzed, the contemporary positivity of at least two of them in the same sample was more common than the detection of only one. Among the most common associations, we found *ermB + tet(M)* (55 cases) and *ermB* + *tet(M)* + *ermF* (31 cases) (see Table S2 for detailed results).

More than 80% of *tet(M)*-positive cases (147/175) were associated with the presence of the conjugative transposon *tet(M)*-tn916.

Interestingly, with the exception of *tet(W)*, a significant correlation between the presence of resistance genes and a dysbiotic vaginal status was noticed (Table 1). In particular, for *ermB*, *ermF*, and *tet(M)*, we found an increasing trend of positivity going from a normal vaginal flora to a condition of BV. Several cases of BV (10/35; 28.5%) were characterized by the contemporary positivity of *ermB + tet(M) + ermF* (Table S2).

Considering only the women enrolled at the first trimester of pregnancy, the presence of *ermB* was associated with a higher BMI (*p* = 0.003; BMI: 24.6 ± 3.9 vs. 22.1 ± 2.4). In this context, it is worth mentioning that no significant relationship was noticed between BMI and the vaginal status (H, I, or BV group; *p* = 0.49). On the other hand, the detection of resistance genes was not related to the age of subjects (data not shown). In agreement with the distribution of BV, we noticed that most cases of positivity for *ermB*, *ermF*, and *tet(M)* were found in women at the first trimester of pregnancy (Table S3).

### 2.3. Correlation between Resistance Genes and Vaginal Microbiota

**Figure 1:**
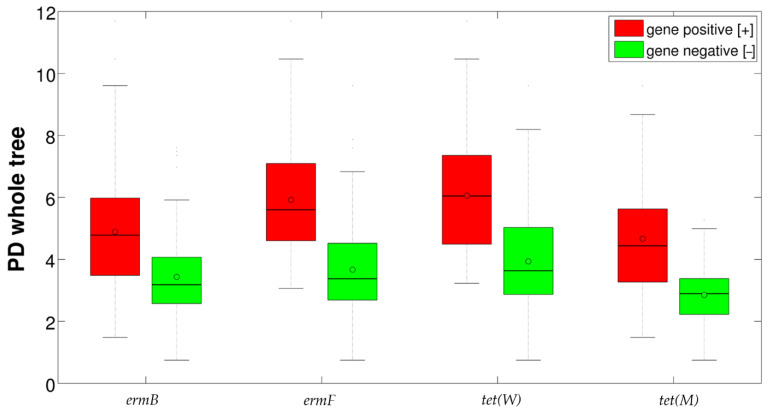
Boxplot of the alpha-diversities (Faith’s phylogenetic diversity metric) of samples positive and negative for the four resistance genes tested. Black lines represent median values; circles represent means.

Alpha-diversity evaluation showed a significant difference (*p* = 0.001) in the biodiversity of vaginal samples, stratified for the positivity/negativity of resistance genes, for all the metrics (i.e., chao1, observed species, phylogenetic diversity (PD) whole tree, Shannon, Good’s coverage). Overall, for all the four genes tested, we observed an increased biodiversity for samples positive to resistance determinants (Figure 1). Stratifying for vaginal status, differences were statistically significant for “H” women for all resistance genes and metrics (data not shown), whereas, when evaluating the combined presence of more resistance genes, a tendency (although not statistically significant) towards an increase of biodiversity following the number of resistance genes detected together was observed (Figure S1).

**Figure 2:**
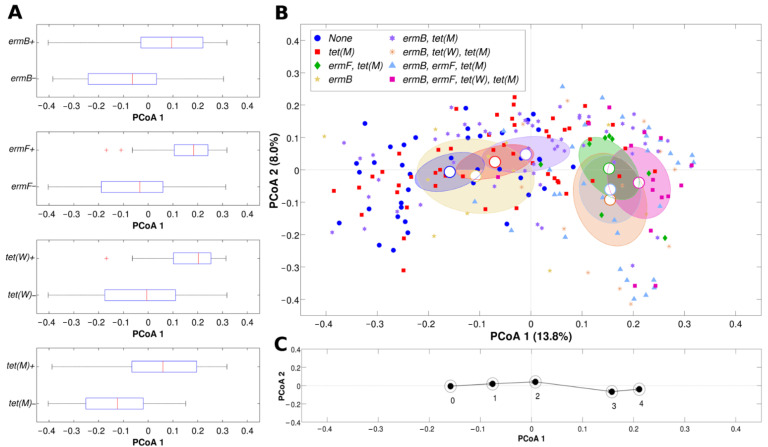
Beta-diversity analysis of microbial profiles according to the presence of the four resistance genes. (**A**) Horizontal boxplots representing the distribution of the first component deriving from principal coordinate analysis (PCoA, unweighted Unifrac distance) for samples positive or negative for each of the four resistance genes tested. (**B**) PCoA of unweighted Unifrac distances among samples; each point represents a sample, centroids are positioned at the average coordinate per group, ellipses are 95% confidence estimates of the standard error of the mean; colors indicate a different combination of the presence of the four resistance genes; only combinations with >1 sample per group were considered; the first and second principal coordinate are represented. (**C**) Plot representing the centroids of the PCoA (unweighted Unifrac) of the samples grouped according to the number of resistance genes; the first and second principal coordinates are represented.

Evaluation of the microbial composition (beta-diversity analysis) confirmed the evidence suggested above, with samples displaying significant differences in composition based on the detection of resistance genes, for both unweighted and weighted Unifrac distances (Figure 2A).

When looking at the single or combined presence of more than one resistance gene per specimen (Figure 2B), we noticed that *ermF* and *tet(M)* seemed to be the determinants contributing most to the separation of the vaginal samples. All samples negative for *ermF* and *tet(M)* fell in the leftmost part of the Principal Coordinates Analysis (PCoA), whereas *tet(M)*-positive samples were in the rightmost part.

Interestingly, at the extreme left of the plot, we found samples negative to all four genes, whereas on the far right we had samples positive for all the resistance determinants, in a sort of ‘microbiota’ trajectory, shifting from left to right with increasing number of resistance genes (Figure 2C).

Overall, resistance genes seemed to be associated with a status of dysbiosis, since in samples positive for at least one resistance determinant, a general decrease in *Lactobacillus* spp. and an increase of BV-related genera (e.g., *Prevotella*, *Gardnerella*) was noticed. Average relative abundances of each taxa showing a significant difference between samples negative or positive for resistance genes are shown in Table 2.

**Figure 3:**
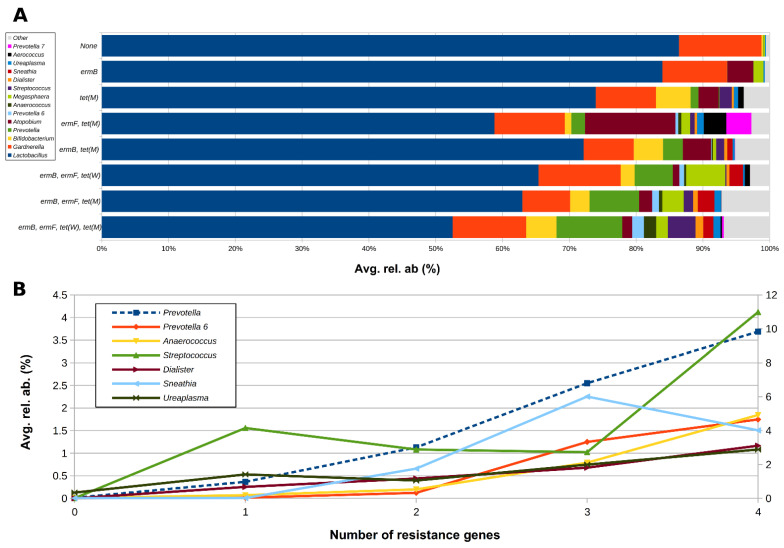
(**A**) Horizontal barplots of the average relative abundance of the main genera constituting the vaginal microbiota of the tested women, grouped according to the combination of the different resistance genes; only combinations with >1 sample per group and only genera with avg. rel. ab. >1% in at least one combination were considered. (**B**) Line plot of the average relative abundance of a selection of genera from the vaginal microbiota, showing an increasing trend with increasing number of resistance genes per sample; for graphical purposes, *Prevotella* abundance (dashed blue line) is represented on the secondary y-axis.

Samples negative for all the analyzed genes showed the highest average abundance of *Lactobacillus* spp. (86.4%) and the lowest of BV-associated taxa, such as *Prevotella*, *Atopobium*, and *Anaerococcus* (Figure 3). In contrast, the abundance of *Lactobacillus* spp. dropped drastically (about 50%) in samples positive for all the four genes. It is worth noting that the levels of *Prevotella, Anaerococcus, Streptococcus, Dialister, Sneathia*, and *Ureaplasma* tended to increase progressively as the number of positive genes per sample increased (Figure 3).

Stratifying for the vaginal status (H, I, or BV), this tendency was confirmed, particularly for the “H” group: *Lactobacillus* tended to decrease in samples positive for the resistance genes, whereas *Prevotella* and *Atopobium* tended to increase (Table S4).

The alterations suggested by analyzing the relative abundance of bacterial genera were confirmed by performing point-biserial correlation between genus-level relative abundances and the presence/absence of resistance genes. As shown in Table 3, the positivity to any resistance gene was negatively correlated to *Lactobacillus* spp. abundance and positively to *Prevotella*, *Dialister*, and *Anaerococcus*. Higher levels of *Atopobium* were associated with the positivity of all genes, with the exception of *tet(W)*, whereas *Sneathia* was positively related to *ermB* and *tet(W)*. A positive association between *Gardnerella*, *ermF*, and *tet(W)* was also found. Interestingly, *Bifidobacterium* was positively associated with both the tetracycline resistance determinants.

Since the positivity to a certain resistance gene could vary over time (Table S3), a survival analysis was performed over the gestation and post-partum weeks in order to further investigate how the bacterial groups identified contributed to the positivity status of women during pregnancy.

**Figure 4:**
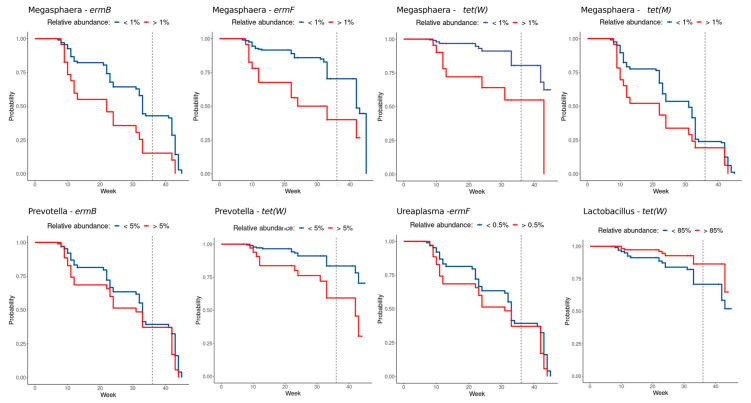
Kaplan-Meier curves of resistance gene positivity. Detection of a resistance gene was exploited as a survival event among samples above (red) or under (blue) bacterial relative abundance thresholds. Crosshairs represent censored observations. All curves reported have a significant log-rank separation (*p*-value < 0.05).

Higher abundances of the *Lactobacillus* genus seem to have a protective role towards the incidence of resistance genes (for *tet(W)* in particular), which appeared less frequently when the vaginal microbiome was dominated by it. In contrast, higher abundances of BV-related bacteria (such as *Megasphaera*, *Prevotella*, and *Ureaplasma*) (Figure 4) show an opposite trend: the higher the bacterial abundance, the higher the probability of manifesting resistance genes. Additional survival plots, showing the probabilities related to other bacterial genera and thresholds, are depicted in Figure S2.

### 2.4. Correlation between BMI and Vaginal Microbiota Composition

In general, only a few significant correlations were observed between BMI (considered only for women during the first trimester of pregnancy) and the composition of the vaginal microbiota. At the phylum level, there was a slightly negative correlation between BMI and Tenericutes abundance (r = −0.24). At the family and genus level, we found that BMI was negatively correlated with the Leptotrichaceae family (r = −0.28) and *Sneathia* genus (r = −0.31) and positively correlated with the *Prevotella* genus (r = 0.24).

## 3. Discussion

In this study we assessed the distribution of selected tetracycline and macrolide resistance genes in the vaginal microbiota of pregnant women at different gestational ages.

In particular, we analyzed the presence of *ermB*, *ermF*, *tet(W)*, and *tet(M)* genes in the vaginal ecosystem of women during the three trimesters of pregnancy and puerperium, deciphering the correlations between the presence of resistance determinants and the abundance of vaginal bacterial taxa.

At first, we found that some resistance genes were very common in the vaginal environment of pregnant women, with the prevalence of *tet(M)* and *ermB* exceeding 55%. Other resistance determinants, namely *ermF* and *tet(W)*, were less common, showing a 25% and 14% prevalence, respectively.

Recently, Roachford and colleagues assessed the cervicovaginal resistome in a cohort of Afro-Caribbean women by means of whole genome shotgun metagenomics. They confirmed that the most abundant resistance determinants are related to tetracyclines (*tet*; about 50%) and macrolides (*erm*; about 15%), with genes encoding for tetracycline-resistant ribosomal protection proteins being the most common [23].

The distribution of resistance genes found in our cohort is similar to the one described for the human gut microbiomes (i.e., fecal samples), with a significantly high prevalence of *ermB* and *tet(M)* genes [19,20,28]. Thus, we can speculate that the gastro-intestinal tract could serve as a reservoir of bacteria-related resistance genes, able to easily reach the vaginal environment by means of microbial translocation [29]. This aspect can partly explain the occurrence of resistance genes during puerperium, since a significant vaginal ‘contamination’ from intestinal-derived microbes occurs during labor and delivery.

Significant data emerged when the presence of resistance genes was related to the vaginal status (i.e., H, I, and BV groups based on Nugent score) and to the bacterial composition of the vaginal microbiota (i.e., 16S rRNA gene sequencing). We noticed that, except for *tet(W)*, the detection of resistance determinants was significantly associated with BV status, with the prevalence of resistance genes increasing along with the worsening of the vaginal dysbiosis (i.e., going from H to I to BV status). Interestingly, we found that a higher number of combined resistance genes (i.e., more than one resistance gene in the same sample) was related to a greater distance from a normal microbiota. Thus, an increased polymicrobism, typical of severe BV conditions [30], led to an easier occurrence of multiple resistance genes at the same moment. In agreement with these results, the presence of resistance genes was more common when, during pregnancy, the conditions of vaginal dysbiosis are more frequent (i.e., first trimester and puerperium) [12,31].

As expected, the positivity of resistance genes was positively related with several BV-related taxa (e.g., *Prevotella*, *Dialister*, *Anaerococcus*, *Atopobium*, and *Gardnerella*) and negatively related to the abundance of vaginal *Lactobacillus* spp. In line with these findings, we demonstrated that a high predominance of *Lactobacillus* spp. in the vaginal environment (>85%) during pregnancy is associated with a lower risk of *tet(W)* gene detection, whereas the presence of several BV-associated bacteria significantly increase, in time, the chance of positivity of one or more resistance determinants (e.g., the presence of *Megasphaera* >1% increases the risk of positivity for all analyzed genes, whereas *Prevotella* >5% significantly increases the risk for *erm(B)* and *tet(W)*).

Even though culture-based approaches will be needed to assess the exact distribution of resistance genes among bacterial genera, we can speculate that each genus is characterized by a different antimicrobial resistance pattern, linked to a different bacterial plasticity and different responses to antibiotic selective pressure.

All these data strengthen the idea that a lactobacilli-dominated microbiota is associated with vaginal eubiosis and wellbeing, whereas a BV condition can negatively affect the women’s health, being a state that broadly correlates with increased risk of infection, disease, and poor reproductive and obstetric outcomes [32].

As indicated by our results (i.e., high association between *tet(M)* and the conjugative transposon *tet(M)*-tn916), macrolide and tetracycline resistance genes can be linked to mobile elements, thus favoring horizontal transfer of resistance determinants from commensal vaginal inhabitants to pathogens [16]. Moreover, during delivery, microbial communities can be transferred from the mother’s vaginal niche to the newborn gut, thus affecting the infant’s microbiome development and future health [33]. Along with microbial transfer, newborns can acquire bacteria-associated resistance genes [22,34]. As previously shown [22], newborns acquire tetracycline antibiotic resistance genes from mothers at birth, especially *tet(M)* and *tet(O)* in case of vaginal delivery.

Although the strongest correlations were found for BV-associated genera, it is worth mentioning that even ‘health-promoting’ microorganisms can harbor resistance determinants. In agreement with our results, it has been shown that resident bifidobacteria can possess genes conferring resistance to tetracyclines [35]. However, it should be remembered that Bifidobacteria are typical beneficial commensals inhabiting the human intestine and are only minority components of the vaginal consortium [36].

It is worth noting that the presence of the *ermB* gene was associated with a higher BMI at the beginning of pregnancy. Moreover, *ermB* was found to be specially correlated with higher vaginal levels of *Prevotella* genus, in turn associated with higher BMI levels. In this context, it has been shown that host obesity significantly increased the diversity of the vaginal microbiota in association with *Prevotella*, whose relative abundances are strongly associated with BV [37]. Thus, an adequate body weight, together with a pre-pregnancy correct dietary intake, seem protective factors against BV condition, for the maintenance of a healthy vaginal flora during pregnancy [10].

In conclusion, we were able to find a sort of ‘vaginal fingerprint’, being different types of microbiota composition associated with peculiar resistance profiles. If a ‘normal’ vaginal ecosystem is poor in or free of resistance genes, a condition of dysbiosis (i.e., BV) is strongly associated with the presence of more than one determinant of antimicrobial resistance. These data could open new perspectives for promoting vaginal health during pregnancy, with the aim of maintaining a lactobacilli-dominated vaginal ecosystem, in turn depleted of antimicrobial resistance genes.

Further studies are needed for a deeper comprehension of the potential origin and ‘sources’ of the antimicrobial resistance genes (e.g., food, water, past use of antibiotics, or microbiome ‘sharing’ with partner) [13,21,38,39]. Future perspectives include (i) collection of detailed and accurate medication history, to find correlations between resistance genes and past use of antimicrobials, (ii) analysis of a large panel of resistance determinants, (iii) bacterial isolation from vaginal swabs and assessment of resistance genes in each single strain, and (iv) study of mother-newborn couples to monitor the dynamics of resistance determinant transfer.

## 4. Materials and Methods

### 4.1. Study Population and Sample Collection

From April 2019, all the Caucasian pregnant women presenting to the Family Advisory Health Centers of Ravenna (Italy) for prenatal care were considered eligible for the study.

Exclusion criteria were the following: (i) age < 18 years; (ii) HIV positivity; (iii) body mass index (BMI) > 33; (iv) medically assisted procreation; (v) use of any antibiotics in the month preceding the sampling; (vi) use of vaginal douches or topical agents in the two weeks before sampling; (vii) presence of uncontrolled chronic diseases (e.g., diabetes, autoimmune disorders, malignancies); (viii) drug addiction or heavy smokers (>15 cigarettes/day). Moreover, women with urogenital infections due to sexually transmitted pathogens (i.e., *Chlamydia trachomatis*, *Neisseria gonorrhoeae*, *Trichomonas vaginalis*, and *Mycoplasma genitalium*), aerobic vaginitis, or symptomatic candidiasis were excluded after the laboratory testing.

Women underwent a clinical visit at different gestational ages (i.e., 9–13 weeks, first trimester; 20–24 weeks, second trimester; 32–34 weeks, third trimester) and during the puerperium (40–55 days after delivery). Demographic data and clinical information were recorded for each patient.

Two vaginal swabs were collected from each woman. The first one (collected by E-swab collection system, Copan, Brescia, Italy) was used for microbiological diagnostic tests and Nugent score assessment. The second one (collected by a sterile cotton bud swab) was employed for microbiota analysis and for the detection of resistance genes (see specific paragraphs below).

Written informed consent was obtained from all subjects, and the study protocol was approved by the Ethics Committee of Romagna (CEROM) (n° 2032 of 21 February 2018). This study was carried out in accordance with the Declaration of Helsinki, following the recommendations of the Ethics Committee.

### 4.2. Microbiological Investigations

A commercial nucleic acid amplification technique (NAAT) was used for the detection of sexually transmitted pathogens (i.e., *C. trachomatis*, *N. gonorrhoeae*, *T. vaginalis*, and *M. genitalium*; Seeplex STI Master Panel 1; Seegene, Seoul, Korea). Microscopic examination and cultures were performed for candidiasis and aerobic vaginitis diagnosis [40,41].

A Gram stain scoring system (Nugent score) was used for a preliminary assessment of the vaginal flora composition [27]. Based on this score, women were grouped as follows: “H” group (normal lactobacilli-dominated microbiota, score 0–3), “I” group (intermediate microbiota, score 4–7), “BV” group (dysbiosis condition, namely bacterial vaginosis, score 8–10) [42].

### 4.3. Detection of Resistance Genes

Nucleic acids were extracted from vaginal swabs by means of the Versant molecular system (Siemens Healthcare Diagnostics, Tarrytown, NY, USA), as previously described [43]. Starting from the obtained eluate, each sample underwent both the detection of resistance genes and the analysis of the vaginal microbiome composition (see paragraph below).

The presence of *ermB*, *ermF*, *tet(W)*, and *tet(M)* genes was assessed by means of in-house end-point PCR assays. *tet(W)* and *tet(M)* genes confer antimicrobial resistance to tetracyclines by encoding for ribosome protection types of tetracycline resistance proteins, whereas *ermB* and *ermF* are methylase-type erythromycin resistance genes, conferring resistance to macrolides [44,45].

A PCR test targeting the conjugative transposon (mobile element) carrying the resistance gene *tet(M)*, namely *tet(M)*-tn916, was performed as well [13].

Each reaction consisted of 45 µL of PCR mix (GoTaq^®^ G2 Master Mix, Promega, Milan, Italy) and 5 µL of target. For each gene, primer sequences, PCR conditions, and amplicon size are reported in detail in Table S5. A sample was considered positive if the PCR test, after 35 cycles, gave an amplicon of the expected size.

Associations between the presence of resistance genes and available variables (e.g., Nugent score, BMI, age) were searched by *t*-test or Chi-square test, where appropriate.

A *p* value < 0.05 was considered as statistically significant.

### 4.4. Microbiota Analysis

The V3–V4 hypervariable regions of the bacterial 16S rRNA gene were amplified, according to the 16S metagenomic sequencing library preparation protocol (Illumina, San Diego, CA, USA). Final indexed libraries were prepared by equimolar (4 nmol/L) pooling, denaturation, and dilution to 6 pmol/L, before loading onto the MiSeq flow cell (Illumina). A 2 × 300 bp paired-end run was used.

Raw sequencing reads were processed, generating a single fragment covering the whole amplicon from the two overlapping pairs, using PandaSeq software (v2.5, [46]), keeping 250–900 base long fragments and filtering out those having more than 25% nucleotides with a Phred score ≤3.

Quality filtering, taxonomy assignments, and diversity analyses of the samples were performed using the QIIME suite (release 1.9.0, [47]). Filtered reads were de-duplicated and de-noised, creating zero-radius Operational Taxonomic Units (zOTUs), using the unoise3 algorithm [48] provided in the usearch pipeline (v. 11.0.667) and discarding those with less than 5 supporting reads. Taxonomic assignment was performed against the SILVA 16S rRNA database (release 132, https://www.arb-silva.de/fileadmin/silva_databases/qiime/Silva_132_release.zip accessed on 25 November 2021) through the RDP classifier at 0.5 confidence [49].

α-diversity evaluation was estimated according to several microbial diversity metrics (i.e., chao1, Shannon index, observed species, Good’s coverage, and Faith’s phylogenetic distance). β-diversity analysis was conducted using both weighted and unweighted Unifrac metrics [50] and through the Principal Coordinate Analysis (PCoA).

Survival analysis was performed on bacterial genera selected as among the most representative from the taxonomic classification, and the detection of positivity to one of the resistance genes in the single samples over time was considered as a censoring event.

Several thresholds of bacterial relative abundance were implemented as time-dependent covariates, which started at value 0 and changed to 1 once the relative abundance of the genus in the sample increased above a certain value. Bacterial thresholds were determined from the mean abundances of all 228 samples and pondering the number of samples that would fall in one of the sides (keeping 30 samples at the first time-point as minimum acceptable quantity). For a better analysis performance, the original four collection points, divided into trimesters and days after delivery, were subdivided into 20 time points according to the weeks of pregnancy and post-partum, with week 36 as an indicative delivery time point.

### 4.5. Statistical Method

Statistical evaluation of α-diversity indices was performed by non-parametric Monte Carlo-based tests through the QIIME pipeline. β-diversity differences were assessed by a permutation test with pseudo F-ratios using the “adonis” function from R package “vegan” (version 2.0-10, [51]). Pairwise relative abundance analysis was performed using a non-parametric Mann–Whitney *U* test with Benjamini-Hochberg False-Discovery Rate correction on the 15 most abundant genera. For comparing relative abundances across multiple categories, we applied a Kruskal-Wallis test, followed by Dunn’s post hoc test for pairwise comparisons.

Correlation between microbial composition at the genus level and presence/absence of each resistance gene was calculated using the point biserial correlation [52], whereas correlation between microbial profiles and BMI was performed using Spearman’s rank-based correlation coefficient. In both cases, only coefficients showing a *p* value of the linear model <0.05 were reported. Statistical evaluations were performed in Matlab (Software version 7.7.0, Natick, MA, USA).

Survival analysis was performed through the RStudio software (version 1.2.1335; R version 3.6.3) using a custom pipeline employing the packages “survival” (v 3.2-3) and “survminer” (v 0.4.9); statistical differences between Kaplan-Meyer curves were determined through a log-rank test.

A *p* value < 0.05 was considered as statistically significant for all analysis.

## Figures and Tables

**Table 1:** Distribution of macrolide and tetracycline resistance genes, stratified for the vaginal status by Nugent score [27]. Statistical analysis was performed by Chi-square test.

Gene	H % (n = 142)	I % (n = 51)	BV % (n = 35)	*p* Value
*ermB*	49.2% (70)	58.8% (30)	74.2% (26)	0.024
*ermF*	19.0% (27)	29.4% (15)	45.7% (16)	0.003
*tet(M)*	71.1% (101)	82.3% (42)	91.4% (32)	0.021
*tet(W)*	14.0% (20)	11.7% (6)	20.0% (7)	0.553

**Table 2:** Bacterial genera statistically different between samples showing presence and absence of each resistance gene. “Gene positive [+]/negative [−]” refers to the average relative abundance of each genus on all samples positive/negative for the specified resistance gene.

Gene	Phylogenetic_Name	Avg. Relative Abundance (%)		Significance ^a^
		Gene Positive [+]	Gene Negative [−]	
*ermB*	*Lactobacillus*	67.1	76.6	*
	*Prevotella*	5.0	0.8	***
	*Atopobium*	2.9	2.6	***
	*Streptococcus*	1.4	1.0	*
*ermF*	*Lactobacillus*	57.2	76.2	***
	*Gardnerella*	11.6	9.5	*
	*Prevotella*	7.3	1.8	***
	*Atopobium*	3.3	2.6	***
	*Streptococcus*	1.9	1.0	*
	*Prevotella 6* ^b^	1.3	0.1	***
	*Anaerococcus*	1.1	0.1	***
*tet(W)*	*Lactobacillus*	57.4	73.7	**
	*Bifidobacterium*	4.8	3.1	**
	*Prevotella*	7.0	2.5	***
	*Atopobium*	1.1	3.0	**
	*Sneathia*	1.5	0.6	**
	*Prevotella 6*	1.6	0.2	***
	*Anaerococcus*	1.5	0.2	**
*tet(M)*	*Lactobacillus*	67.8	83.0	***
	*Bifidobacterium*	4.2	0.8	**
	*Prevotella*	4.0	0.4	***
	*Atopobium*	3.4	0.7	***
	*Streptococcus*	1.6	0.0	***

^a^*p*-value of Mann-Whitney *U*-test, with Benjamini-Hochberg FDR correction. ***: *p* < 0.001; **: *p* < 0.01; *: *p* < 0.05. ^b^ *Prevotella* genus in SILVA database (release 132) was split into multiple groups according to sequence similarity. *Prevotella 6* group includes the following species: *Prevotella bergensis*, *P. colorans*, *P. corporis*, and *P. salivae*, plus other non-species characterized strains and some uncultured bacteria.

**Table 3:** Correlation between the relative abundance of vaginal bacterial genera and presence of macrolide and tetracycline resistance genes. Only genera with an average relative abundance >0.4% were reported. “--” indicates that the *p*-value of the linear model for correlation calculation was >0.05.

Genera	Resistance Gene			
	*ermB*	*ermF*	*tet(W)*	*tet(M)*
*Lactobacillus*	−0.136	−0.239	−0.165	−0.185
*Gardnerella*	--	0.046	0.074	--
*Bifidobacterium*	--	--	0.051	0.123
*Prevotella*	0.264	0.304	0.199	0.192
*Atopobium*	0.016	0.029	−0.062	0.100
*Streptococcus*	0.029	0.066	--	0.104
*Sneathia*	0.205	--	0.093	--
*Alloscardovia*	--	--	--	0.105
*Ureaplasma*	--	0.175	--	--
*Dialister*	0.154	0.200	0.153	0.201
*Prevotella 6*	0.099	0.306	0.271	0.125
*Aerococcus*	--	0.035	0.003	0.076
*Anaerococcus*	0.067	0.280	0.300	0.125

## Data Availability

Raw sequencing data of 16S rRNA gene are available at NCI SHort-reads Archive (SRA, https://www.ncbi.nlm.nih.gov/sra/ accessed on 25 November 2021) with BioProject accession number PRJNA766806. Other data presented in this study are available in the Supplementary Materials.

## Supplementary Materials

The following are available online at https://www.mdpi.com/article/10.3390/pathogens10121546/s1, Table S1: Vaginal status, stratified by the gestational age, Table S2: Prevalence of resistance genes, Table S3: Distribution of macrolide and tetracycline resistance genes, stratified for the gestational age, Table S4: Bacterial genera statistically different (*p* < 0.05, Mann-Whitney U-test) between samples, Table S5: List of primers and PCR conditions used for the detection of resistance genes, Figure S1: Alpha diversity boxplots for the four resistance genes combination, Figure S2: All statistically significant survival curves
