# Supplementary material for: Distribution of ermB, ermF, tet(W), and tet(M) Resistance Genes in the Vaginal Ecosystem of Women during Pregnancy and Puerperium
Source: Pathogens. 2021 Nov 26;10(12):1546. doi: 10.3390/pathogens10121546 (PMC8705968; doi:10.3390/pathogens10121546)

SUPPLEMENTARY MATERIAL

Distribution of *ermB, ermF, tet(W)*, and *tet(M)* resistance genes in the vaginal ecosystem of women during pregnancy and puerperium

Marco Severgnini[1^], Tania Camboni[1^], Camilla Ceccarani[1], Sara Morselli[2], Alessia Cantiani[2], Sara Zagonari[3], Giulia Patuelli[3], Maria Federica Pedna[4], Vittorio Sambri[2,4], Claudio Foschi[2]*, Clarissa Consolandi[1], Antonella Marangoni[2]

[1]Institute of Biomedical Technologies – National Research Council, Segrate, Milan, Italy

[2]Microbiology, DIMES, University of Bologna, Bologna, Italy

[3]Family Advisory Health Centres, Ravenna, Italy

[4]Unit of Microbiology, Greater Romagna Hub Laboratory, Pievesestina di Cesena, Italy

^Marco Severgnini and Tania Camboni equally contributed to this work

*Corresponding author: Claudio Foschi (claudio.foschi2@unibo.it)

Supplementary tables

Table S1. Vaginal status, stratified by the gestational age……………………………………………………………………………3

Table S2. Prevalence of resistance genes……………………………………………………………………………………………………4

Table S3. Distribution of macrolide and tetracycline resistance genes, stratified for the gestational age……..5

Table S4. Bacterial genera statistically different (*p*<0.05, Mann-Whitney U-test) between samples……………6

Table S5. List of primers and PCR conditions used for the detection of resistance genes……………………………..8

Supplementary figures

Figure S1. Alpha diversity boxplots for the four resistance genes combination……………………………………………9

Figure S2. All statistically significant survival curves……………………………………………………………………………………10

Table S1. Vaginal status, stratified by the gestational age.

Time point	H (n=142)	I (n=51)	BV (n=35)
1st trimester	33 (23.2%)	26 (51.0%)	13 (37.1%)
2nd trimester	47 (33.1%)	10 (19.6%)	6 (17.1%)
3rd trimester	52 (36.6%)	7 (13.7%)	4 (11.4%)
puerperium	10 (7.1%)	8 (15.7%)	12 (34.3%)

Table S2. Prevalence of resistance genes. Prevalence for all cases and those showing a BV vaginal status is shown.

**Resistance genes**	**n° of cases (tot=228)**	**BV cases (n=35)**
None	39 (17.1%)	1 (2.8%)
tet(M)	51 (22.3%)	6 (17.1%)
ermB	10 (4.4%)	1 (2.8%)
ermF	0 (0.0%)	0 (0.0%)
tet(W)	1 (0.4%)	0 (0.0%)
ermB + tet(M)	55 (24.1%)	7 (20.0%)
ermF + tet(M)	8 (3.5%)	2 (5.7%)
tet(M) + tet(W)	1 (0.4%)	0 (0.0%)
ermB + erm(F)	1 (0.4%)	1 (2.8%)
ermB + tet(W)	0 (0.0%)	0 (0.0%)
ermF + tet(W)	1 (0.4%)	0 (0.0%)
ermB + ermF + tet(M)	31 (13.6%)	10 (28.6%)
ermB + tet(M) + tet(W)	13 (5.7%)	4 (11.4%)
ermB + ermF + tet(W)	1 (0.4%)	0 (0.0%)
ermF + tet(M) + tet(W)	1 (0.4%)	1 (2.8%)
ermB + ermF + tet(M) + tet(W)	15 (6.6%)	2 (5.7%)

Table S3. Distribution of macrolide and tetracycline resistance genes, stratified for the gestational age.

Time point	***ermB*** **(n=126)**	***ermF*** **(n=58)**	***tet(M)*** **(n=175)**	***tet(W)*** **(n=33)**
1st trimester	44 (34.9%)	23 (39.6%)	55 (31.4%)	11 (33.4%)
2nd trimester	33 (26.2%)	11 (19.0%)	46 (26.3%)	8 (24.2%)
3rd trimester	27 (21.4%)	13 (22.4%)	47 (26.8%)	8 (24.2%)
Puerperium	22 (17.5%)	11 (19.0%)	27 (15.4%)	6 (18.2%)

Table S4. **Bacterial genera statistically different (*p*<0.05, Mann-Whitney U-test) between samples.** Presence and absence of each resistance gene, stratified according to women's vaginal status, is shown. For each gene and category, the number of samples is indicated.

Gene	Status	N+[a]	N-[b]	Genus	Avg. rel. ab (%) Gene positive [+]	Gene negative [-]	Direction[c]
ermB	BV	26	9	*Prevotella*	13.0	2.6	↑
				Megasphaera	7.9	1.3	↑
				Sneathia	5.5	0.0	↑
				Prevotella 7	0.0	3.4	↓
				DNF00809	1.0	0.1	↑
	I	30	21	*Prevotella*	6.4	2.5	↑
	H	70	72	*Prevotella*	1.5	0.1	↑
				Atopobium	2.3	0.1	↑
				Streptococcus	0.1	0.3	↓
ermF	BV	16	19	*Sneathia*	6.2	2.3	↑
				Ureaplasma	1.0	0.1	↑
				Prevotella 6	3.3	0.6	↑
				DNF00809	1.3	0.3	↑
				Fastidiosipila	1.1	0.3	↑
	I	15	36	*Lactobacillus*	55.5	79.2	↓
				Prevotella	9.0	3.0	↑
				Atopobium	8.1	2.0	↑
				Streptococcus	4.9	0.6	↑
	H	27	115	*Lactobacillus*	74.3	84.8	↓
				Bifidobacterium	5.2	3.1	↑
				Prevotella	2.8	0.3	↑
				Atopobium	1.3	1.1	↑
tet(M)	BV	32	3	*Gardnerella*	18.13	76.18	↓
				Dialister	1.45	0.02	↑
				Prevotella 6	2.02	0.00	↑
	I	42	9	--	--	--	--
	H	101	41	*Lactobacillus*	79.87	90.06	↓
				Bifidobacterium	4.89	0.00	↑
				Prevotella	1.12	0.03	↑
				Atopobium	1.64	0.00	↑
tet(W)	BV	7	28	*Megasphaera*	13.95	4.30	↑
				Dialister	1.99	1.17	↑
				Prevotella 6	4.51	1.18	↑
				Aerococcus	0.33	1.72	↓
				Fastidiosipila	1.29	0.48	↑
	I	6	45	*Lactobacillus*	51.74	74.93	↓
				Bifidobacterium	9.91	2.76	↑
	H	20	122	*Lactobacillus*	72.33	84.53	↓
				Bifidobacterium	4.96	3.24	↑
				Prevotella	3.21	0.41	↑
				Atopobium	0.49	1.28	↓
				Anaerococcus	1.30	0.08	↑

[a] Number of samples positive to each resistance gene per each vaginal status

[b] Number of samples negative to each resistance gene per each vaginal status

[c] ↑ indicates an increase in gene positive samples with respect to gene negative ones. ↓ indicates the opposite

Table S5. List of primers and PCR conditions used for the detection of resistance genes.

Gene	Primers	PCR conditions	Amplicon size	Reference
ermB	5’-GAAAAGGTACTCAACCAAATA-3’<br>5’-AGTAACGGTACTTAAATTGTTTAC-3’	95°C for 10 min; 35 cycles of 94°C for 1 min, 54°C for 1 min and 72°C for 1 min; 72°C for 7 min	639 bp	Milanovic, 2017 [a]
ermF	5’-CGGGTCAGCCTTTACTATTG-3’<br>5’-GGACCTACCTCATAGACAAG-3’	95°C for 10 min; 35 cycles of 94°C for 1 min, 48°C for 1 min and 72°C for 1 min; 72°C for 7 min	466 bp	Sirichoat, 2020 [b]
tet(M)	5’-ACCCGTATACTATTTCATGCACT-3’<br>5’-CCTTCCATAACCGCATTTTG-3’	95°C for 10 min; 35 cycles of 94°C for 1 min, 48°C for 1 min and 72°C for 2 min; 72°C for 7 min	1115 bp	Milanovic, 2017 [a]
tet(W)	5’-GAGAGCCTGCTATATGCCAGC-3’<br>5’-GGGCGTATCCACAATGTTAAC-3’	95°C for 10 min; 35 cycles of 94°C for 1 min, 62°C for 1 min and 72°C for 1 min; 72°C for 7 min	168 bp	Milanovic, 2017 [a]
***tet(M)*-tn916**	5’-TACTACCGGTGAACCTGTTTGCCA-3’<br>5’-TTTAGCCAGCGGTATCAACGAAGC-3’	95°C for 10 min; 35 cycles of 94°C for 1 min, 55°C for 1 min and 72°C for 1 min; 72°C for 7 min	472 bp	Jeters, 2009 [c]

a Milanović V., Osimani A., Aquilanti L., et al. (2017). Occurrence of antibiotic resistance genes in the fecal DNA of healthy omnivores, ovo-lacto vegetarians and vegans. Mol Nutr Food Res, 61(9).

b Sirichoat A., Flórez A.B., Vázquez Let al. (2020). Antibiotic susceptibility profiles of lactic acid bacteria from the human vagina and genetic basis of acquired resistances. Int J Mol Sci, 21(7):2594

c Jeters R. T., Rivera A. J., Boucek L.M., et al. (2009). Antibiotic resistance genes in the vaginal microbiota of primates not normally exposed to antibiotics. Microb Drug Resist, 15(4):309-315

Figure S1. Alpha diversity boxplots for the four resistance genes combination. Boxplot of the alpha-diversities (Faith's phylogenetic diversity metric) of samples grouped according to the combination of four resistance genes tested. Red lines represent median values, whereas blue ones represent means; yellow dots are the values for the single sample in each category; only combinations with >1 sample per group were considered.

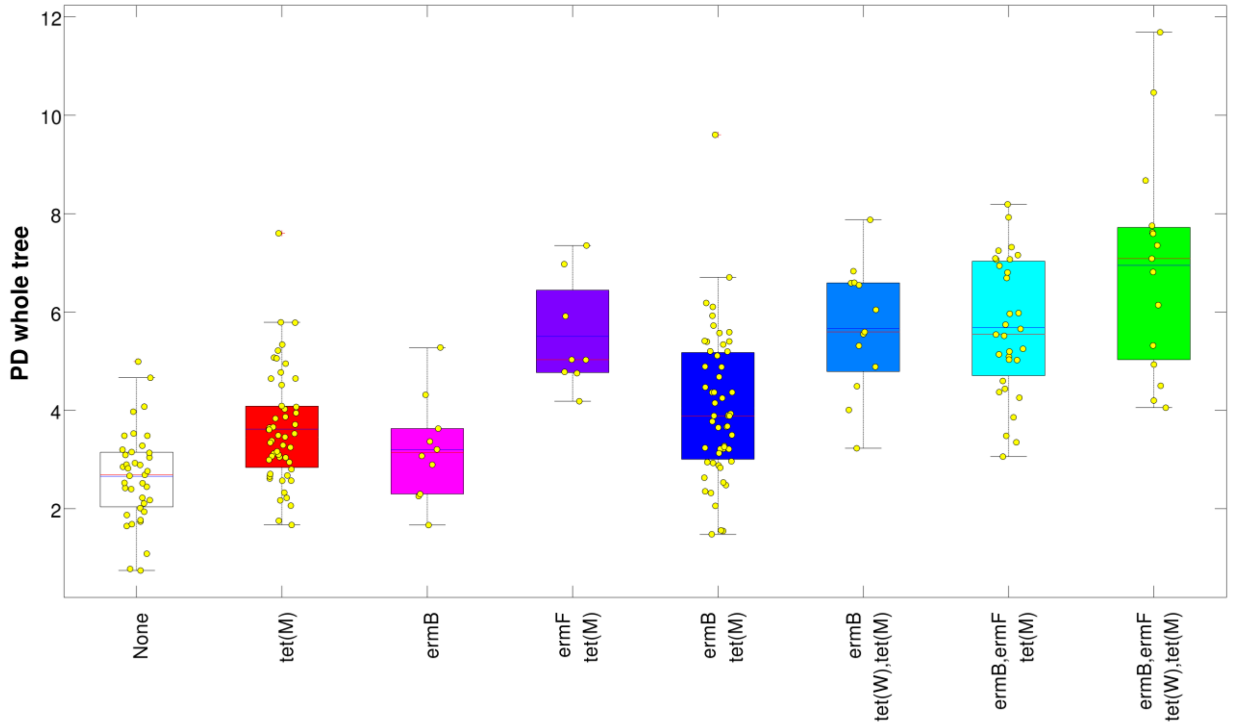

Figure S2. All statistically significant survival curves. For any combination of genus-resistance gene, statistically significant differences (log-rank *p*-value <0.05) are shown. For each genus-gene, the lowest abundance threshold for which the test was significant is reported.

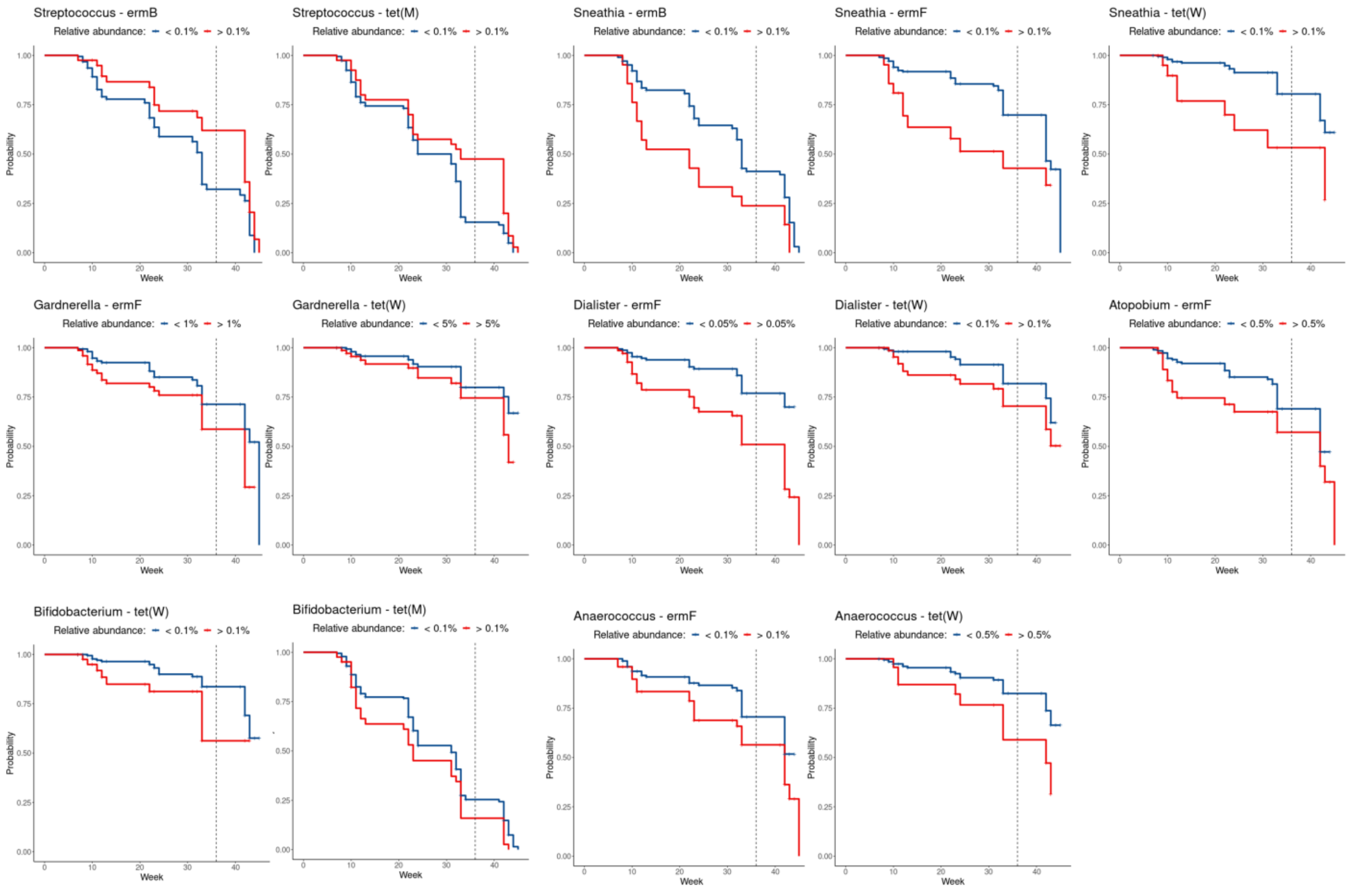

Supplement: Supplementary file 1 [file pathogens-10-01546-s001.zip › pathogens-1453592-supplementary.pdf]
